# Preoperative estimated glomerular filtration rate as a significant predictor of long-term outcomes after coronary artery bypass grafting in Japanese patients

**DOI:** 10.1007/s11748-013-0306-5

**Published:** 2013-08-15

**Authors:** Satoru Domoto, Osamu Tagusari, Yoshitsugu Nakamura, Hideaki Takai, Yoshimasa Seike, Yujiro Ito, Yuko Shibuya, Fumiaki Shikata

**Affiliations:** 1 Department of Cardiovascular Surgery, NTT Medical Center Tokyo, 5-9-22 Higashigotanda, Shinagawa-ku, Tokyo, 141-8625 Japan; 2 Department of Hypertension and Nephrology, NTT Medical Center Tokyo, 5-9-22 Higashigotanda, Shinagawa-ku, Tokyo, 141-8625 Japan; 3 Division of Cardiovascular Surgery, Ehime University, Shitsukawa, Toon-shi, Ehime 791-0295 Japan

**Keywords:** Estimated glomerular filtration rate, Long-term outcomes, Coronary artery bypass grafting, Chronic kidney disease

## Abstract

**Purposes:**

The aim of this retrospective study was to investigate the effect of chronic kidney disease (CKD) on outcomes after coronary artery bypass grafting (CABG), and to determine whether preoperative estimated glomerular filtration rate (eGFR) can be a predictor of long-term outcomes after CABG.

**Methods:**

486 Japanese patients who underwent isolated CABG between December 2000 and August 2010 were evaluated. Preoperative eGFR was estimated by the Japanese equation according to guidelines from the Japanese Society of Nephrology. We defined CKD as a preoperative eGFR of less than 60 ml/min/1.73 m^2^. 203 patients had CKD (CK group) and 283 patients did not (N group).

**Results:**

During a mean observation time of 53 months, the overall survival rate was significantly lower in the CK group than in the N group (*p* = 0.0044). Similarly, the CK group had significantly more unfavorable results with regard to freedom from cardiac death, major adverse cardiovascular and cerebrovascular events (MACCE), and hemodialysis. Using multivariate analyses, preoperative eGFR was an independent predictor of all-cause mortality (HR 0.983; *p* = 0.026), cardiac mortality (HR 0.963; *p* = 0.006), and incidence of MACCE (HR 0.983; *p* = 0.002).

**Conclusions:**

The CK group had significantly more unfavorable outcomes than the N group. Preoperative eGFR was an independent predictor of long-term outcomes after CABG in Japanese patients.

## Introduction

Severe renal dysfunction, in particular, which requires dialysis, has been identified as a risk factor for adverse outcomes after coronary artery bypass grafting (CABG) [1–3]. Chronic kidney disease (CKD) that does not require dialysis, which has been defined according to levels of serum creatinine or estimated glomerular filtration rate (eGFR) calculated by the Cockcroft–Gault equation, has also been identified as a predictor of poor outcomes after CABG [4–11]. It has been reported recently that eGFR calculated using the Modification of Diet in Renal Disease (MDRD) study equation is a more accurate marker of renal function than either creatinine clearance or eGFR calculated by the Cockroft–Gault equation. Thus, the calculation of eGFR in this way could aid in the diagnosis of mild renal impairment, even in patients with normal or nearly normal creatinine levels [12]. A limited number of studies have defined CKD according to eGFR calculated using the MDRD study equation, assessed the impact of CKD on long-term outcomes after CABG, and determined whether preoperative eGFR is a predictor of long-term outcomes after CABG [13–15]. It has been also reported that the revised Japanese equation is more accurate for the Japanese population than the MDRD study equation using the previous Japanese Society of Nephrology Chronic Kidney Disease Initiative [16]. In this study, we calculated eGFR and defined CKD using the Japanese equation according to guidelines from the Japanese Society of Nephrology. The aim of this retrospective study was to investigate the effect of CKD on early and long-term outcomes after CABG in Japanese patients, and to determine whether preoperative eGFR is a predictor of long-term outcomes after CABG.

## Patients and methods

Between December 2000 and April 2010, 527 consecutive Japanese patients underwent isolated CABG at our institution. Excluding 41 patients who underwent preoperative hemodialysis (HD) or previous cardiac surgery, 486 patients were evaluated.

### Definition of CKD

We defined CKD as a preoperative eGFR of less than 60 ml/min/1.73 m^2^ according to guidelines from the National Kidney Foundation [17–19]. eGFR was calculated using the Japanese equitation according to guidelines from the Japanese Society of Nephrology [16]: eGFR (ml/min/1.73 m^2^) = 194 × (serum creatinine [mg/dl])^−1.094^ × (age [years])^−0.287^ × 0.739 (in the case of female patients). Note that all our patients were Japanese. Preoperative eGFR was calculated at admission.

### Definition of end points

The end points studied overall death, cardiac death, incidence of major adverse cardiovascular and cerebrovascular events (MACCE) and introduction to HD. Cardiac death included deaths caused by myocardial infarction, heart failure, or sudden death. Follow-up information was obtained from each patient’s hospital records, interviews at the time of outpatient visit, telephone calls and from referring physicians.

### Surgical technique

Since October 2001, we have performed off-pump CABG (OPCAB) for patients requiring coronary artery revascularization as the first-line treatment. The internal thoracic artery (ITA), gastroepiploic artery (GEA), and radial artery (RA) were harvested in all cases with the skeletonization technique. The basic strategy for myocardial revascularization was in situ grafting of bilateral ITAs to the left coronary system, with complementary RA or saphenous vein graft (SVG). In most patients, the in situ left ITA was grafted to the left anterior descending (LAD) artery, and the in situ right ITA was grafted to the circumflex branches. RA and SVG were used for aorto-coronary bypass to revascularize the posteroinferior wall. However, in cases of critical coronary stenosis, GEA was used as an in situ graft. If it was necessary to revascularize several vessels in the left coronary system, and to avoid manipulation of the ascending aorta with highly arteriosclerosis, the in situ left ITA was anastomosed to the LAD artery, the RA graft was anastomosed to the in situ right ITA in an end-to-side fashion, and this was grafted to the circumflex branches and right coronary artery.

### Statistical analysis

Normally distributed continuous data were presented as mean (SD). Discrete variables were compared with the *χ* ^2^ test, and continuous variables were compared with the Mann–Whitney test. The Kaplan–Meier method was used for determining the overall survival, freedom from cardiac death, freedom from MACCE, and freedom from HD, while the log-rank test was applied for statistical comparison. Potential independent predictors of outcomes were identified by univariate Cox regression analysis. All univariable predictors were then entered in a stepwise manner into a multivariable Cox regression analysis, with entry and retention set at a significance level of *p* < 0.05. Hazard ratios (HRs) were reported with 95 % confidence intervals (CIs). All statistical analyses were carried out using Dr. SPSS II (SPSS, Inc., Chicago, IL, USA) for Windows.

## Results

### Preoperative data

Patients’ preoperative characteristics are shown in Table 1: 203 (42 %) patients had CKD (CK group) and 283 (58 %) patients did not (N group). Mean serum creatinine and eGFR were 1.24 ± 0.50 mg/dl and 45.9 ± 10.3 ml/min/1.73 m^2^, respectively, in the CK group, and 0.76 ± 0.13 mg/dl and 77.1 ± 13.8 ml/min/1.73 m^2^, respectively, in the N group. There were no significant differences in characteristics between the 2 groups except for age, proteinuria, preoperative hemoglobin, history of stroke, and old myocardial infarction (OMI). Age was higher in the CK group (71.7 ± 8.5 years) than in the N group (65.7 ± 10.2 years, *p* < 0.01), and preoperative hemoglobin was lower in the CK group (12.3 ± 1.8 g/dl) than in the N group (13.3 ± 1.5 g/dl, *p* < 0.01). Proteinuria, stroke, and OMI were more frequent in the CK group than in the N group (proteinuria: 22 vs. 10 %, *p* < 0.01; stroke: 21 vs. 13 %, *p* = 0.02; OMI: 48 vs. 37 %, *p* = 0.02).

**Table 1:** Preoperative characteristics

	CK (*n* = 203)	N (*n* = 283)	*p* value
Age (year)	71.7 ± 8.5	65.7 ± 10.2	<0.01
Male	152 (74 %)	234 (83 %)	0.04
Creatinine (mg/dl)	1.24 ± 0.50	0.76 ± 0.13	<0.01
eGFR (ml/min/1.73 m^2^)	45.9 ± 10.3	77.1 ± 13.8	<0.01
Proteinuria	45 (22 %)	29 (10 %)	<0.01
Hypertension	144 (71 %)	186 (66 %)	NS
Dyslipidemia	80 (40 %)	126 (45 %)	NS
Diabetes	105 (52 %)	133 (47 %)	NS
Stroke	42 (21 %)	36 (13 %)	0.02
PAD	23 (11 %)	20 (7 %)	NS
Smoking history	122 (60 %)	189 (67 %)	NS
Hemoglobin (g/dl)	12.3 ± 1.8	13.3 ± 1.5	<0.01
Cardiac data			
Prior PCI	21 (10 %)	35 (12 %)	NS
AMI <3 weeks ago	122 (60 %)	189 (67 %)	NS
OMI	97 (48 %)	104 (37 %)	0.02
Emergency	39 (12 %)	46 (16 %)	NS
NYHA III–IV	38 (19 %)	42 (15 %)	NS
Low EF (<30 %)	22 (11 %)	20 (7 %)	NS
Coronary artery disease			
LMT disease	46 (23 %)	62 (22 %)	NS
3 vessels	122 (60 %)	182 (64 %)	NS
2 vessels	69 (34 %)	88 (31 %)	NS
1 vessels	12 (6 %)	13 (5 %)	NS

*eGFR* estimated glomerular filtration rate, *PAD* peripheral artery disease, *PCI* percutaneous catheter intervention, *AMI* acute myocardial infarction, *OMI* old myocardial infarction, *EF* ejection fraction, *LMT* left main trunk

### Intraoperative data

Intraoperative data are shown in Table 2. There were no significant differences between the 2 groups except for RA use and transfusion. RA grafts were used less often in the CK group (37 vs. 48 %, *p* = 0.02), and transfusion was used more frequently in the CK group than in the N group (65 vs. 44 %, *p* < 0.01).

**Table 2:** Intraoperative data

	CK (*n* = 203)	N (*n* = 283)	*p* value
OPCAB	185 (91 %)	233 (82 %)	NS
Operation time (min)	311 ± 73	320 ± 76	NS
No. distal anastomosis	3.2 ± 1.2	3.2 ± 1.2	NS
Grafts			
Single ITA	53 (26 %)	87 (30 %)	NS
Bilateral ITAs	149 (73 %)	196 (70 %)	NS
All arterial grafts use	157 (77 %)	232 (82 %)	NS
RA use	75 (37 %)	136 (48 %)	0.02
GEA use	51 (25 %)	76 (27 %)	NS
SVG use	46 (23 %)	51 (18 %)	NS
Composite grafting	37 (18 %)	37 (13 %)	NS
Transfusion	131 (65 %)	124 (44 %)	<0.01

*OPCAB* off-pump coronary artery grafting, *ITA* internal thoracic artery, *RA* radial artery, *GEA* gastroepiploic artery, *SVG* saphenous vein graft

### Postoperative outcomes

Postoperative outcomes are shown in Table 3. Hospital death occurred in the case of 3 patients in the CK group. Two of these deaths were caused by low output syndrome, and one was caused by pneumonia. There was no special treatment for CKD in the intraoperative and postoperative management, and there were no significant differences between the 2 groups with regard to the number of hospital deaths, postoperative course, and complications.

**Table 3:** Postoperative outcomes

	CK (*n* = 203)	N (*n* = 283)	*p* value
Hospital death	3 (1.5 %)	0 (0 %)	NS
Postoperative course			
Intubation >24 h	14 (6.9 %)	17 (6.0 %)	NS
ICU stay (day)	3.4 ± 7.1	2.6 ± 3.6	NS
Hospital stay (day)	15 ± 14	14 ± 11	NS
Complications			
Re-exploration	1 (0.5 %)	1 (0.4 %)	NS
Perioperative MI	3 (1.5 %)	2 (0.7 %)	NS
Stroke	1 (0.5 %)	1 (0.5 %)	NS
Respiratory failure	12 (5.9 %)	8 (2.8 %)	NS
Perioperative HD	2 (1.0 %)	0 (0 %)	NS
Late tamponade	1 (0.5 %)	2 (0.7 %)	NS
Atrial fibrillation	52 (26 %)	60 (21 %)	NS
Ventricular arrhythmia	4 (2.0 %)	2 (0.7 %)	NS
Mediastinitis	2 (1.0 %)	2 (0.7 %)	NS
SSI	4 (2.0 %)	5 (1.8 %)	NS

*ICU* intensive care unit, *HD* hemodialysis, *SSI* surgical site infection

### Long-term outcomes

**Fig. 1:**
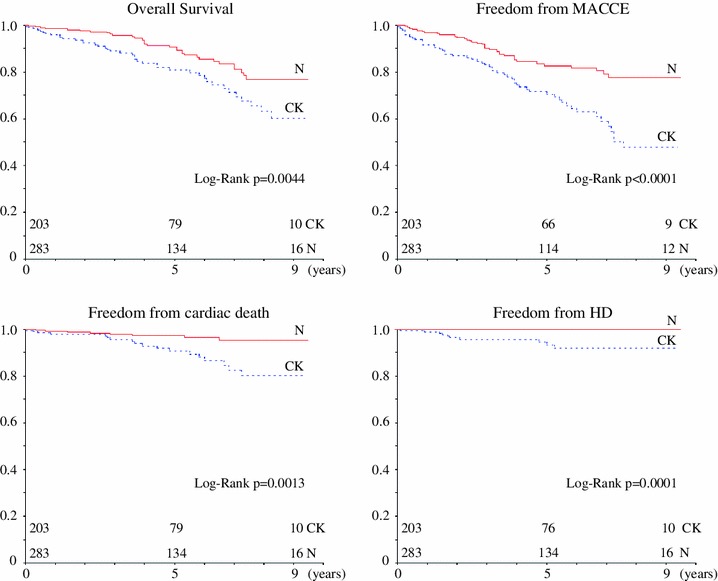
Kaplan–Meier curves show that the CK group has significantly more unfavorable long-term outcomes than N group

Long-term outcomes are shown in Table 4. During the mean follow-up period of 53 ± 33 months, cardiac deaths occurred in the case of 18 patients in the CK group and 8 patients in the N group. Three of these deaths in the CK group were caused by acute myocardial infarction (AMI), and 15 were caused by heart failure. No patients were introduced to HD in the N group, whereas 10 patients were introduced to HD in the CK group. Kaplan–Meier curves (Fig. 1) showed that compared to the N group, the CK group had significantly poorer overall survival (at 5 and 9 years, 81 and 60 % vs. 91 and 77 %, respectively, *p* = 0.0044), freedom from cardiac death (at 5 and 9 year, 91 and 80 % vs. 97 and 95 %, respectively, *p* = 0.0013), freedom from MACCE (at 5 and 9 year, 71 and 48 % vs. 83 and 78 %, respectively, *p* < 0.0001), and freedom from HD (at 5 and 9 years, 93 and 92 % vs. 100 and 100 %, respectively, *p* = 0.0001).

**Table 4:** Long-term outcomes (mean 53 months)

	CK (*n* = 203)	N (*n* = 283)
MACCE		
AMI	5 (2.5 %)	8 (2.8 %)
Heart failure	24 (12 %)	6 (2.1 %)
PCI	17 (8.4 %)	16 (5.7 %)
Cerebrovascular events	11 (5.4 %)	15 (7.4 %)
Late death		
Cardiac death	18 (8.9 %)	8 (2.8 %)
Cerebrovascular death	7 (3.4 %)	4 (1.4 %)
Non cardiac death	15 (7.4 %)	21 (7.4 %)
HD	10 (4.9 %)	0 (0 %)

*MACCE* major adverse cardiovascular and cerebrovascular events

Predictors of long-term outcomes were identified by univariate and multivariate Cox regression analyses. Predictors of all-cause mortality identified by univariate and multivariate Cox regression analyses are shown in Table 5. Univariate analysis identified age, serum creatinine, preoperative eGFR (considered as a continuous variable), proteinuria, dyslipidemia, stroke, peripheral artery disease (PAD), smoking history, preoperative hemoglobin (considered as a continuous variable), NYHA III–IV, low ejection fraction (EF <30 %), OMI, AMI, emergency, transfusion, RA use, GEA use, and SVG use as predictors of long-term mortality. Serum creatinine and eGFR were mathematically related. However, in this cohort, when eGFR was used as a predictor, creatinine provided no additional prognostic information. Therefore, eGFR was used in subsequent forward conditional multivariable models. All predictors identified by univariate analysis, except serum creatinine, were used in the multivariate analysis. Preoperative eGFR (HR 0.983; 95 % CI 0.969–0.998; *p* = 0.026) and transfusion (HR 2.227; 95 % CI 1.143*–*4.338; *p* = 0.019) proved to be independent predictors of all-cause mortality. Exclusion of age (which is a component of eGFR) did not change in the result of this model. Predictors of cardiac mortality, incidence of MACCE, and introduction to HD identified by multivariate Cox regression analysis are shown in Table 6. Preoperative eGFR proved to be an independent predictor of cardiac mortality (HR 0.963; 95 % CI 0.938–0.990; *p* = 0.006) and incidence of MACCE (HR 0.983; 95 % CI 0.972–0.994; *p* = 0.002). Again, exclusion of age did not alter this result. Preoperative hemoglobin and emergency were independent predictors of cardiac mortality, where PAD was an independent predictor of the incidence of MACCE.

**Table 5:** Predictors of long-term mortality by univariate and multivariate Cox regression analysis (all-cause mortality)

	Univariate analysis		Multivariate analysis	
	HR (95 % CI)	*p* value	HR (95 % CI)	*p* value
Age	1.069 (1.039–1.100)	<0.001	1.026 (0.993–1.060)	0.118
Male	1.109 (0.619–1.989)	0.729		
Creatinine	2.009 (1.516–2.906)	<0.001		
eGFR	0.972 (0.960–0.985)	<0.001	0.983 (0.969–0.998)	0.026
Proteinuria	1.846 (1.058–3.219)	0.031	1.116 (0.601–2.071)	0.728
Hypertension	1.050 (0.643–1.715)	0.844		
Dyslipidemia	0.527 (0.309–0.899)	0.019	0.730 (0.417–1.278)	0.271
Diabetes	1.367 (0.860–2.172)	0.186		
Stroke	2.309 (1.349–3.954)	0.002	1.704 (0.893–3.251)	0.106
PAD	2.996 (1.641–5.472)	<0.001	1.278 (0.632–2.585)	0.495
Smoking history	1.029 (0.640–1.653)	0.907		
Hemoglobin	0.773 (0.670–0.892)	<0.001	0.970 (0.819–1.149)	0.724
NYHA III–IV	3.611 (2.203–5.918)	<0.001	1.473 (0.636–3.411)	0.366
Low EF (<30 %)	4.131 (2.360–7.232)	<0.001	1.978 (1.001–3.910)	0.05
OMI	1.751 (1.100–2.788)	0.018	1.392 (0.834–2.324)	0.206
Prior PCI	1.208 (0.619–2.358)	0.588		
AMI <3 weeks ago	4.010 (2.231–7.208)	<0.001	1.290 (0.524–3.176)	0.58
Emergency	3.421 (2.111–5.546)	<0.001	1.402 (0.632–3.113)	0.406
Pump	0.806 (0.422–1.539)	0.514		
Operation time	0.997 (0.994–1.001)	0.117		
Transfusion	4.362 (2.500–7.609)	<0.001	2.227 (1.143–4.338)	0.019
RA use	0.589 (0.355–0.975)	0.039	1.020 (0.545–1.908)	0.952
GEA use	0.541 (0.314–0.931)	0.027	0.735 (0.393–1.372)	0.333
SVG use	2.031 (1.257–3.281)	0.004	1.289 (0.663–2.503)	0.454
Bilateral ITAs	1.221 (0.738–2.020)	0.436		

*HR* hazard ratio

**Table 6:** Multivariate analysis

	HR (95 % CI)	*p* value
Cardiac mortality		
eGFR	0.963 (0.938–0.990)	0.006
Hemoglobin	0.711 (0.534–0.946)	0.019
Emergency	2.978 (1.030–8.609)	0.044
Incidence of MACCE		
eGFR	0.983 (0.972–0.994)	0.002
PAD	2.331 (1.293–4.203)	<0.01

In 10 patients introduced to HD, characteristics are shown in Table 7. The mean serum creatinine and preoperative eGFR were 2.32 ± 1.02 mg/dl (range 1.45–4.36 mg/dl) and 25.2 ± 9.51 ml/min/1.73 m^2^ (range 11.8–39.5 ml/min/1.73 m^2^). The mean periods from CABG to HD were 29.3 ± 22.2 months (range 1–63 months). Three patients were in CKD stage 3 (30–60 ml/min/1.73 m^2^) and two of them exacerbated diabetic nephropathy. Predictors of introduction to HD in patients with CKD identified by univariate and multivariate Cox regression analyses are shown in Table 8. Preoperative eGFR of less than 30 ml/min/1.73 m^2^ (HR 71.04; 95 % CI 7.037–717.2; *p* < 0.001), proteinuria (HR 7.565; 95 % CI 1.221–46.86; *p* = 0.030), diabetes (HR 7.844; 95 % CI 1.026–59.97; *p* = 0.047), and preoperative hemoglobin (HR 0.371; 95 % CI 0.190–0.727; *p* = 0.004) were identified as independent predictors of introduction to HD by multivariate analysis. When preoperative eGFR is considered as a continuous variable, similar significant results were obtained (HR 0.828; 95 % CI 0.743–0.924; *p* < 0.001).

**Table 7:** Characteristics

Age (year)	69.0 ± 10.4
Male	8
Creatinine (mg/dl)	2.32 ± 1.02
eGFR (ml/min/1.73 m^2^)	25.2 ± 9.5
CKD Stage 5	2
CKD Stage 4	5
CKD Stage 3	3
Periods from CABG to HD (months)	29.3 ± 22.2
Proteinuria	8
Hypertension	8
Dyslipidemia	1
Diabetes	8
Stroke	5
PAD	3
Smoking history	3
Hemoglobin (g/dl)	12.3 ± 1.8
NYHA III–IV	3
Low EF (<30 %)	2
3 vessels disease	5
2 vessels disease	5
OPCAB	10
No. distal anastomosis	2.8 ± 1.0
Bilateral ITAs	8
RA use	0
GEA use	3
SVG use	4

**Table 8:** Predictors of the introduction to HD by univariate and multivariate Cox regression analysis in patients with CKD

	Univariate analysis		Multivariate analysis	
	HR (95 % CI)	*p* value	HR (95 % CI)	*p* value
eGFR <30	30.10 (7.723–117.3)	<0.001	71.04 (7.037–717.2)	<0.001
Proteinuria	21.91 (4.625–103.8)	<0.001	7.565 (1.221–46.86)	0.03
Diabetes	4.969 (1.048–23.56)	0.043	7.844 (1.026–59.97)	0.047
Stroke	5.212 (1.485–18.30)	0.01	3.019 (0.651–14.01)	0.158
PAD	4.127 (1.065–16.00)	0.04	0.553 (0.091–3.350)	0.519
Hemoglobin	0.497 (0.331–0.749)	<0.001	0.371 (0.190–0.727)	0.004

## Discussion

Glomerular filtration rate is accepted as the best overall measure of renal function. GFR is measured as the urinary or plasma clearance of a filtration marker such as inulin. However, measuring the clearance of inulin is complex and expensive. In routine clinical practice, eGFR calculated from the MDRD study equation is the most accurate marker of renal function [12] and revised Japanese equation is more accurate for the Japanese population than the MDRD study equation [16].

Several studies have reported poor outcomes in patients with renal dysfunction after CABG, but long-term data are scarce. In addition, most studies, that have shown an association between preoperative renal dysfunction and a higher incidence of morbidity and mortality after CABG, have concentrated on patients with elevated serum creatinine or decreased eGFR calculated by the Cockcroft–Gault equation [1–11]. In order to evaluate renal function accurately in Japanese patients, this study defined CKD as a preoperative eGFR of less than 60 ml/min/1.73 m^2^, which was calculated from the Japanese equation. The results confirm the major impact of CKD on the long-term outcomes after CABG.

The association between CKD and poor outcomes after CABG in patients who are not on dialysis has multiple possible explanations. Firstly, increased risk may be attributed to a multitude of concomitant factors seen in patients with CKD, including advanced age, low preoperative hemoglobin, history of OMI, and stroke (Table 1). CKD is associated with the processes of these diseases, which are themselves determinants of poor outcomes [20]. Advanced age, low preoperative hemoglobin correlated with renal function, and low ejection fraction are known to be associated with an increased risk of morbidity and mortality following CABG [21–23]. History of stroke is also known to be a predictor of cerebrovascular events after cardiac surgery requiring cardiopulmonary bypass [24]. Using univariate analysis, the current study confirmed that these preoperative factors are predictors of long-term outcomes after CABG (Table 5). Secondly, CKD is a powerful independent risk factor for cardiovascular disease. This may reflect the increased inflammation and oxidative stress associated with reduced renal function. In addition, kidney dysfunction may be associated with many other physical changes including high levels of homocysteine, hyperuricemia, hypercalcemia, and uremia, all of which have detrimental cardiovascular effects [25, 26]. Thirdly, it has been reported that patients with CKD have a greater frequency of triple vessel disease and left main involvement, when compared to patients without CKD [13]. This indicates that patients with CKD may have more extensive coronary disease preoperatively than patients without CKD. The deleterious consequences of CKD may lead to a global reduction in oxygen supply to the myocardium, due to severe damage to epicardial coronary macrovessels and depressed coronary reserve secondary to microvessel disease [27]. In our study, there were no significant differences between the 2 groups in the frequency of triple vessel disease and left main trunk disease, or the number of distal anastomoses. However, it is possible that these patients still have coronary microvessel disease.

The surgical strategy used for CABG, including graft selection, may be an important predictor of outcomes for patients with CKD. Recent reviews have demonstrated that in patients with CKD, OPCAB is less deleterious than conventional CABG, and the use of total arterial grafts, especially bilateral ITAs have better outcomes after CABG [4, 28, 29]. We generally use the bilateral ITAs in these patients; with this approach, the RA is preserved as an arteriovenous fistula for vascular access in the future (Table 2). As a result, all arterial grafts are used less frequently than SVG in patients with CKD, when compared to patients without CKD. In this study, there were no differences between the 2 groups in early outcomes (Table 3); however, SVG use was identified as a predictor of poorer long-term outcomes by univariate analysis (Table 5). Therefore, we have confirmed the importance of using in situ bilateral ITAs, considering the advantage of long-term graft patency. In addition, it may be necessary to more clearly define preoperative eGFR criteria for RA use and evaluating the risk of HD. Considering this result (Table 8), we propose that RA should not be used in CKD patients with a preoperative eGFR of less than 30 ml/min/1.73 m^2^, proteinuria, diabetes, or low hemoglobin to prevent introduction to HD in the future.

The significantly increased risk of all-cause death, cardiac death, and the incidence of MACCE in patients with CKD indicates that postoperative treatment for cardiorenal protection is necessary after CABG. It has been reported that CKD is associated with decreased utilization of key cardiovascular medications such as aspirin, β-blockers, statins, angiotensin-converting enzyme inhibitors, and angiotensin receptor blockers [30]. Since reduction in renal function is a powerful predictor of adverse outcomes, preservation of renal function is directly linked with secondary prevention of cardiovascular events after CABG and in other cardiovascular disease settings.

### Limitations

This study has several limitations. Firstly, our study population was small, and the follow-up period was short. Secondly, preoperative eGFR was based on a single measurement at admission. However, eGFR may fluctuate, particularly in patients with unstable hemodynamics and varying medical therapy. Thirdly, no data were available on medications taken after discharge, because the patients received postoperative treatment from different hospitals. However, most patients were discharged with aspirin, β-blocker, statin, and angiotensin-converting enzyme inhibitor or angiotensin receptor blocker therapy.

## Conclusions

Our study clearly demonstrates that CKD has an unfavorable impact on long-term outcomes after CABG and preoperative eGFR is a significant predictor of long-term outcomes after CABG. These results lead us to recommend the incorporation of preoperative eGFR into the risk assessments of long-term outcomes after CABG.

## References

[1] Liu JY, Birkmeyer NJO, Sanders JH, Morton JR, Henriques HF, Lahey SJ (2000). Risk of mortality in dialysis patients undergoing coronary artery bypass surgery. Circulation.

[2] Labrousse L, de Vincentiis C, Madonna F, Deville C, Roques X, Baudet E (1999). Early and long-term results of coronary artery grafts in patients with dialysis dependent renal failure. Eur J Cardiothorac Surg.

[3] Khaitan L, Sutter FP, Goldmand SM (2000). Coronary artery bypass grafting in patients who requiring long-term dialysis. Ann Thorac Surg.

[4] Nakayama Y, Sakata R, Ura M, Itho T (2003). Long-term results of coronary artery bypass grafting in patients with renal insufficiency. Ann Thorac Surg.

[5] Devbhandari MP, Duncan AJ, Grayson AD, Fabri BM, Keenan DJM, Bridgewater B (2006). Effect of risk-adjusted, non-dialysis-dependent renal dysfunction on mortality and morbidity following coronary artery bypass surgery: a multi-centre study. Eur J Cardiothorac Surg.

[6] Weerasinghe A, Hornick P, Smith P, Taylor K, Ratnatunga C (2000). Coronary artery bypass grafting in non-dialysis dependent mild-to-moderate renal dysfunction. J Thorac Cardiovasc Surg.

[7] Rao V, Weisel RD, Buth KJ, Cohen G, Borger MA, Shiono N (1997). Coronary artery bypass grafting in patients with non-dialysis-dependent renal insufficiency. Circulation.

[8] Zakeri R, Freemantle N, Barnett V, Lipkin GW, Bonser RS, Graham TR (2005). Relation between mild renal dysfunction and outcomes after coronary artery bypass grafting. Circulation.

[9] van Straten AHM, Hamad MAS, van Zundert AAJ, Martens EJ, Schönberger JPAM, de Wolf AM (2009). Preoperative renal function as a predictor of survival after coronary artery bypass grafting: comparison with a matched general population. J Thorac Cardiovasc Surg.

[10] Holzmann MJ, Hammar N, Ahnve S, Nordqvist T, Pehrsson K, Ivert T (2007). Renal insufficiency and long-term mortality and incidence of myocardial infarction in patients undergoing coronary artery bypass grafting. Eur Heart J.

[11] Qiang Z, Chang-Sheng M, Shao-Ping N, Xin D, Qiang L, Jun-Ping K (2007). Prevalence and impact of renal insufficiency on clinical outcomes of patients undergoing coronary revascularization. Circulation J.

[12] Stevens LA, Coresh J, Greene T, Levey AS (2006). Assessing kidney function: measured and estimated glomerular filtration rate. N Engl J Med.

[13] Hillis GS, Croal BL, Buchnan KG, El-Shafei H, Gibson G, Jeffrey RR (2006). Renal function and outcome from coronary artery bypass grafting. Impact on mortality after a 2.3-year follow-up. Circulation.

[14] Cooper WA, O’Brien SM, Thourani VH, Guyton RA, Bridges CR, Szczech LA (2006). Impact of renal dysfunction on outcomes of coronary artery bypass surgery: results from the society of thoracic surgeons national adult cardiac database. Circulation.

[15] Kangasniemi O, Mahar MAA, Rasinaho E, Satomaa A, Tiozzo V, Lepojärvi M (2008). Impact of estimated glomerular filtration rate on the 15-year outcome after coronary artery bypass surgery. Eur J Cardiothorac Surg.

[16] Matuo S, Imai E, Horio M, Yasuda Y, Tomita K, Nitta K (2009). Revised equations for estimated GFR from serum creatinine in Japan. Am J Kidney Dis.

[17] National Kidney Foundation (2002). K/DOQI clinical practice guidelines for chronic kidney disease: evaluation, classification, and stratification. Am J Kidney Dis.

[18] Levey AS, Coresh J, Balk E, Kausz AT, Levin A, Steffes MW (2003). National kidney foundation practice guidelines for chronic kidney diseases: evaluation, classification, and stratification. Ann Intern Med.

[19] Levey AS, Eckardt KU, Tsukamoto Y, Levin A, Coresh J, Rossert J (2005). Definition and classification of chronic kidney disease: a position statement from Kidney Disease: improving Global Outcomes (KDIGO). Kidney Int.

[20] Sarnak MJ, Levey AS, Schoolwerth AC, Corech J, Culleton B, Hamm LL (2003). Kidney disease as a risk factor for development of cardiovascular disease: a statement from the American Heart Association councils on kidney in cardiovascular disease, high blood pressure research, clinical cardiology, and epidemiology and prevention. Circulation.

[21] Stamou SC, Dangas G, Dullum MKC, Pfister AJ, Boyce SW, Bafi AS (2000). Beating heart surgery in octogenarians: perioperative outcome and comparison with younger age groups. Ann Thorac Surg.

[22] Bell ML, Grunwald GK, Baltz JH, McDonald GO, Bell MR, Grover FL (2008). Does preoperative hemoglobin independently predict short-term outcomes after coronary artery bypass graft surgery?. Ann Thorac Surg.

[23] Topkara VK, Cheema FH, Kesavaramanujam S, Mercando ML, Cheema AF, Namerow PB (2005). Coronary artery bypass grafting in patients with low ejection fraction. Circulation.

[24] Redmond JM, Greene PS, Goldsborough MA, Cameron DE, Stuart RS, Sussman MS (1996). Neurologic injury in cardiac surgical patients with a history of stroke. Ann Thorac Surg.

[25] Anavekar NS, McMurray JJ, Velazques EJ, Solomon SD, Kober L, Rouleau JL (2004). Relation between renal dysfunction and cardiovascular outcomes after myocardial infarction. N Engl J Med.

[26] Jardine AG (2001). Cardiovascular complications of renal diseases. Heart.

[27] Wizemann V (1996). Coronary artery disease in dialysis patients. Nephron.

[28] Sajja LR, Mannam G, Chakravarthi RM, Sompalli S, Naidu SK, Somaraju B (2007). Coronary artery bypass grafting with or without cardiopulmonary bypass in patients with preoperative non-dialysis dependent renal insufficiency: a randomized study. J Thorac Cardiovasc Surg.

[29] Kinoshita T, Asai T, Murakami Y, Hiramatsu N, Suzuki T, Kambara A (2010). Efficacy of bilateral internal thoracic artery grafting in patients with chronic kidney disease. Ann Thorac Surg.

[30] Gibney EM, Casebeer AW, Schooley LM, Cunningham F, Grover FL, Bell MR (2005). Cardiovascular medication use after coronary bypass surgery in patients with renal dysfunction: a National Veterans Administration study. Kidney Int.

